# Supplementary material for: Neonicotinoids impact bumblebee colony fitness in the field; a reanalysis of the UK’s Food & Environment Research Agency 2012 experiment
Source: PeerJ. 2015 Mar 24;3:e854. doi: 10.7717/peerj.854 (PMC4375969; doi:10.7717/peerj.854)
Supplement: Supplemental Information 1 [file peerj-03-854-s001.docx]

Model outputs from IBM SPSS Statistics 21. Each model contained three explanatory factors: site; concentration of pesticide residue in nectar or pollen; number of bees in the colony at the start of the experiment. Response variable was either colony weight gain or number of queens produced (the latter run using negative binomial errors).

**Colony weight gain**

a) Effects of thiamethoxam in nectar, with pesticide residue levels below the LOD assumed to be zero:

| **Parameter Estimates** | | | | | | | |
| --- | --- | --- | --- | --- | --- | --- | --- |
| Parameter | B | Std. Error | 95% Wald Confidence Interval | | Hypothesis Test | | |
|  |  |  | Lower | Upper | Wald Chi-Square | df | Sig. |
| (Intercept) | 329.889 | 129.8264 | 75.434 | 584.344 | 6.457 | 1 | .011 |
| [Site=1] | 248.334 | 122.2961 | 8.638 | 488.030 | 4.123 | 1 | .042 |
| [Site=2] | 377.796 | 180.8274 | 23.381 | 732.211 | 4.365 | 1 | .037 |
| [Site=3] | 0^a^ | . | . | . | . | . | . |
| Thia-nectar | -108.580 | 59.3406 | -224.885 | 7.726 | 3.348 | 1 | .067 |
| No. bees | 5.114 | 4.5211 | -3.748 | 13.975 | 1.279 | 1 | .258 |

b) Effects of clothianidin in nectar, with pesticide residue levels below the LOD assumed to be zero:

| **Parameter Estimates** | | | | | | | |
| --- | --- | --- | --- | --- | --- | --- | --- |
| Parameter | B | Std. Error | 95% Wald Confidence Interval | | Hypothesis Test | | |
|  |  |  | Lower | Upper | Wald Chi-Square | df | Sig. |
| (Intercept) | 304.396 | 83.0534 | 141.614 | 467.177 | 13.433 | 1 | .000 |
| [Site=1] | 264.281 | 79.9784 | 107.526 | 421.036 | 10.919 | 1 | .001 |
| [Site=2] | 596.605 | 163.7444 | 275.672 | 917.538 | 13.275 | 1 | .000 |
| [Site=3] | 0^a^ | . | . | . | . | . | . |
| No. bees | 5.523 | 3.7938 | -1.912 | 12.959 | 2.120 | 1 | .145 |
| Cloth-nectar | -2476.301 | 804.4566 | -4053.007 | -899.596 | 9.475 | 1 | .002 |

c) Effects of thiamethoxam in pollen, with pesticide residue levels below the LOD assumed to be zero:

| **Parameter Estimates** | | | | | | | |
| --- | --- | --- | --- | --- | --- | --- | --- |
| Parameter | B | Std. Error | 95% Wald Confidence Interval | | Hypothesis Test | | |
|  |  |  | Lower | Upper | Wald Chi-Square | df | Sig. |
| (Intercept) | 419.097 | 133.2125 | 158.005 | 680.189 | 9.898 | 1 | .002 |
| [Site=1] | 195.035 | 131.8874 | -63.460 | 453.529 | 2.187 | 1 | .139 |
| [Site=2] | 241.770 | 154.6865 | -61.410 | 544.950 | 2.443 | 1 | .118 |
| [Site=3] | 0^a^ | . | . | . | . | . | . |
| No. bees | 2.930 | 4.8394 | -6.555 | 12.415 | .367 | 1 | .545 |
| Thia-pollen | -240.589 | 117.8909 | -471.651 | -9.527 | 4.165 | 1 | .041 |

d) Effects of thiamethoxam in nectar, with pesticide residue levels below the LOD assumed to be equal to the LOD:

| **Parameter Estimates** | | | | | | | |
| --- | --- | --- | --- | --- | --- | --- | --- |
| Parameter | B | Std. Error | 95% Wald Confidence Interval | | Hypothesis Test | | |
|  |  |  | Lower | Upper | Wald Chi-Square | df | Sig. |
| (Intercept) | 424.400 | 136.0018 | 157.842 | 690.959 | 9.738 | 1 | .002 |
| [Site=1] | 148.805 | 128.0245 | -102.118 | 399.728 | 1.351 | 1 | .245 |
| [Site=2] | 273.032 | 176.1309 | -72.178 | 618.242 | 2.403 | 1 | .121 |
| [Site=3] | 0^a^ | . | . | . | . | . | . |
| No. bees | 6.257 | 4.6739 | -2.904 | 15.417 | 1.792 | 1 | .181 |
| Thia-nectar | -115.389 | 65.1617 | -243.103 | 12.326 | 3.136 | 1 | .077 |

e) Effects of clothianidin in nectar, with pesticide residue levels below the LOD assumed to be equal to the LOD:

| **Parameter Estimates** | | | | | | | |
| --- | --- | --- | --- | --- | --- | --- | --- |
| Parameter | B | Std. Error | 95% Wald Confidence Interval | | Hypothesis Test | | |
|  |  |  | Lower | Upper | Wald Chi-Square | df | Sig. |
| (Intercept) | 423.932 | 135.5808 | 158.199 | 689.666 | 9.777 | 1 | .002 |
| [Site=1] | 145.623 | 127.3554 | -103.989 | 395.235 | 1.307 | 1 | .253 |
| [Site=2] | 349.290 | 205.2490 | -52.990 | 751.571 | 2.896 | 1 | .089 |
| [Site=3] | 0^a^ | . | . | . | . | . | . |
| No. bees | 5.501 | 4.6669 | -3.646 | 14.648 | 1.389 | 1 | .239 |
| Cloth-nectar | -1812.768 | 1000.3178 | -3773.355 | 147.819 | 3.284 | 1 | .070 |

f) Effects of thiamethoxam in pollen, with pesticide residue levels below the LOD assumed to be equal to the LOD:

| **Parameter Estimates** | | | | | | | |
| --- | --- | --- | --- | --- | --- | --- | --- |
| Parameter | B | Std. Error | 95% Wald Confidence Interval | | Hypothesis Test | | |
|  |  |  | Lower | Upper | Wald Chi-Square | df | Sig. |
| (Intercept) | 630.911 | 163.8730 | 309.726 | 952.096 | 14.823 | 1 | .000 |
| [Site=1] | 151.845 | 122.4174 | -88.089 | 391.779 | 1.539 | 1 | .215 |
| [Site=2] | 164.333 | 129.3857 | -89.258 | 417.924 | 1.613 | 1 | .204 |
| [Site=3] | 0^a^ | . | . | . | . | . | . |
| No. bees | 3.597 | 4.5941 | -5.407 | 12.602 | .613 | 1 | .434 |
| Thia-pollen | -444.641 | 175.8445 | -789.290 | -99.992 | 6.394 | 1 | .011 |

**Queen production**

g) Effects of thiamethoxam in nectar, with pesticide residue levels below the LOD assumed to be zero:

| **Parameter Estimates** | | | | | | | |
| --- | --- | --- | --- | --- | --- | --- | --- |
| Parameter | B | Std. Error | 95% Wald Confidence Interval | | Hypothesis Test | | |
|  |  |  | Lower | Upper | Wald Chi-Square | df | Sig. |
| (Intercept) | 2.337 | .7285 | .909 | 3.764 | 10.289 | 1 | .001 |
| [Site=1] | .844 | .5937 | -.319 | 2.008 | 2.022 | 1 | .155 |
| [Site=2] | 1.123 | .9808 | -.799 | 3.045 | 1.311 | 1 | .252 |
| [Site=3] | 0^a^ | . | . | . | . | . | . |
| No. bees | .032 | .0281 | -.023 | .087 | 1.289 | 1 | .256 |
| Thia-nectar | -.367 | .2832 | -.922 | .188 | 1.677 | 1 | .195 |

h) Effects of clothianidin in nectar, with pesticide residue levels below the LOD assumed to be zero:

| **Parameter Estimates** | | | | | | | |
| --- | --- | --- | --- | --- | --- | --- | --- |
| Parameter | B | Std. Error | 95% Wald Confidence Interval | | Hypothesis Test | | |
|  |  |  | Lower | Upper | Wald Chi-Square | df | Sig. |
| (Intercept) | 2.686 | .7512 | 1.214 | 4.159 | 12.789 | 1 | .000 |
| [Site=1] | .904 | .5864 | -.245 | 2.053 | 2.377 | 1 | .123 |
| [Site=2] | 2.225 | 1.1096 | .050 | 4.400 | 4.022 | 1 | .045 |
| [Site=3] | 0^a^ | . | . | . | . | . | . |
| No. bees | .013 | .0294 | -.045 | .071 | .197 | 1 | .657 |
| Cloth-nectar | -10.207 | 4.4834 | -18.995 | -1.420 | 5.183 | 1 | .023 |

i) Effects of thiamethoxam in pollen, with pesticide residue levels below the LOD assumed to be zero:

| **Parameter Estimates** | | | | | | | |
| --- | --- | --- | --- | --- | --- | --- | --- |
| Parameter | B | Std. Error | 95% Wald Confidence Interval | | Hypothesis Test | | |
|  |  |  | Lower | Upper | Wald Chi-Square | df | Sig. |
| (Intercept) | 2.467 | .7250 | 1.046 | 3.888 | 11.581 | 1 | .001 |
| [Site=1] | .944 | .5970 | -.226 | 2.114 | 2.501 | 1 | .114 |
| [Site=2] | 1.045 | .7392 | -.404 | 2.494 | 1.998 | 1 | .158 |
| [Site=3] | 0^a^ | . | . | . | . | . | . |
| No. bees | .025 | .0277 | -.029 | .079 | .805 | 1 | .370 |
| Thia-pollen | -1.240 | .5587 | -2.335 | -.145 | 4.923 | 1 | .026 |

j) Effects of thiamethoxam in nectar, with pesticide residue levels below the LOD assumed to be equal to the LOD:

| **Parameter Estimates** | | | | | | | |
| --- | --- | --- | --- | --- | --- | --- | --- |
| Parameter | B | Std. Error | 95% Wald Confidence Interval | | Hypothesis Test | | |
|  |  |  | Lower | Upper | Wald Chi-Square | df | Sig. |
| (Intercept) | 2.474 | .7767 | .951 | 3.996 | 10.143 | 1 | .001 |
| [Site=1] | .713 | .5752 | -.415 | 1.840 | 1.535 | 1 | .215 |
| [Site=2] | .805 | .9384 | -1.034 | 2.644 | .736 | 1 | .391 |
| [Site=3] | 0^a^ | . | . | . | . | . | . |
| No. bees | .033 | .0284 | -.023 | .088 | 1.335 | 1 | .248 |
| Thia-nectar | -.304 | .3243 | -.940 | .331 | .881 | 1 | .348 |

k) Effects of clothianidin in nectar, with pesticide residue levels below the LOD assumed to be equal to the LOD:

| **Parameter Estimates** | | | | | | | |
| --- | --- | --- | --- | --- | --- | --- | --- |
| Parameter | B | Std. Error | 95% Wald Confidence Interval | | Hypothesis Test | | |
|  |  |  | Lower | Upper | Wald Chi-Square | df | Sig. |
| (Intercept) | 2.902 | .7977 | 1.339 | 4.466 | 13.238 | 1 | .000 |
| [Site=1] | .739 | .5735 | -.385 | 1.863 | 1.660 | 1 | .198 |
| [Site=2] | 1.873 | 1.0748 | -.234 | 3.979 | 3.036 | 1 | .081 |
| [Site=3] | 0^a^ | . | . | . | . | . | . |
| No. bees | .014 | .0297 | -.044 | .073 | .236 | 1 | .627 |
| Cloth-nectar | -9.682 | 4.8886 | -19.264 | -.101 | 3.923 | 1 | .048 |

l) Effects of thiamethoxam in pollen, with pesticide residue levels below the LOD assumed to be equal to the LOD:

| **Parameter Estimates** | | | | | | | |
| --- | --- | --- | --- | --- | --- | --- | --- |
| Parameter | B | Std. Error | 95% Wald Confidence Interval | | Hypothesis Test | | |
|  |  |  | Lower | Upper | Wald Chi-Square | df | Sig. |
| (Intercept) | 3.399 | 1.0097 | 1.420 | 5.378 | 11.331 | 1 | .001 |
| [Site=1] | .764 | .5758 | -.365 | 1.892 | 1.760 | 1 | .185 |
| [Site=2] | .577 | .6652 | -.727 | 1.880 | .752 | 1 | .386 |
| [Site=3] | 0^a^ | . | . | . | . | . | . |
| No. bees | .024 | .0290 | -.033 | .081 | .682 | 1 | .409 |
| Thia-pollen | -1.828 | 1.0244 | -3.836 | .179 | 3.186 | 1 | .074 |
